# Stroke survivors’ long-term participation in paid employment

**DOI:** 10.3233/WOR-230037

**Published:** 2024-03-08

**Authors:** Winke van Meijeren-Pont, Judith M. van Velzen, Gerard Volker, Henk J. Arwert, Jorit J.L. Meesters, Arend J. de Kloet, Coen A.M. van Bennekom, Thea P.M. Vliet Vlieland, Sietske J. Tamminga, Daniëlla M. Oosterveer, on behalf of the SCORE-study group

**Affiliations:** a Basalt Rehabilitation, Leiden/The Hague, The Netherlands; b Department of Orthopaedics, Rehabilitation, and Physical Therapy, Leiden University Medical Center, Leiden, The Netherlands; c Department of Research and Development, Heliomare Rehabilitation Center, Wijk aan Zee, The Netherlands; d Amsterdam UMC, University of Amsterdam, Coronel Institute of Occupational Health, Amsterdam Public Health Research Institute, Amsterdam, The Netherlands; e Department of Rehabilitation Medicine, Haaglanden Medical Center, The Hague, The Netherlands; f Kenniscentrum Health Innovation, Haagse Hogeschool, The Hague, The Netherlands

**Keywords:** Return to work, rehabilitation, vocational rehabilitation, community participation, personal satisfaction, international classification of functioning, disability and health

## Abstract

**BACKGROUND:**

Knowledge on long-term participation is scarce for patients with paid employment at the time of stroke.

**OBJECTIVE:**

Describe the characteristics and the course of participation (paid employment and overall participation) in patients who did and did not remain in paid employment.

**METHODS:**

Patients with paid employment at the time of stroke completed questions on work up to 30 months after starting rehabilitation, and the Utrecht Scale for Evaluation of Rehabilitation-Participation (USER-P, Frequency, Restrictions and Satisfaction scales) up to 24 months. Baseline characteristics of patients with and without paid employment at 30 months were compared using Fisher’s Exact Tests and Mann-Whitney U Tests. USER-P scores over time were analysed using Linear Mixed Models.

**RESULTS:**

Of the 170 included patients (median age 54.2 interquartile range 11.2 years; 40% women) 50.6% reported paid employment at 30 months. Those returning to work reported at baseline more working hours, better quality of life and communication, were more often self-employed and in an office job. The USER-P scores did not change statistically significantly over time.

**CONCLUSION:**

About half of the stroke patients remained in paid employment. Optimizing interventions for returning to work and achieving meaningful participation outside of employment seem desirable.

## 1 Introduction

Stroke is a common and serious medical condition [1] often leading to impairments in physical and emotional functioning, cognition, and communication [2–4]. This can negatively influence participation in society [5].

Regarding the course of participation on the longer term after stroke, the literature is scarce. Nevertheless, the relatively few available studies with a longer duration of follow-up showed that, despite improvements in particular in the first year, a considerable proportion of patients with stroke still experience restrictions in various aspects of participation on the longer term [6–9]. These restrictions in participation include the domain employment [9]: approximately a quarter of the patients is younger than 65 years at the time of stroke and thus part of the labour force [8]. A review of Treger et al. [10] demonstrated differences between countries in the proportion of stroke patients that return to work. This difference ranged from 14% in Germany to 73% in France and Portugal. Other reviews also mentioned wide ranges of return to work: 4.0–90.9% with a pooled summary estimate of 67.4% two years post stroke [9], 11% 3 months after rehabilitation to 85% 7 years post stroke [11], or 0% 0–3 years post stroke to 100%, with an average of 44% [12, 13]. These differences may not only reflect different international differences such as retirement age or social security systems but may also be related to the inclusion of different stroke populations (population-based, hospital-based, rehabilitation-based), differences in the definition or the assessment of employment status, and differences in the follow-up duration.

Overall it must be noted that most studies on the course of employment status report on one specific time point after stroke, usually not beyond one year, and do not describe the course of returning to work over time. Moreover, most of the studies did not report on aspects of participation other than return to paid employment, whereas participation in other meaningful activities is very important as well, both for patients who do and do not return to work.

Given that knowledge gap, the aim of the present study was to describe the long-term employment outcomes and overall participation in a Dutch cohort of stroke patients who received multidisciplinary rehabilitation and who had paid employment at the time of stroke. More specifically, the study aimed, in patients with paid employment at the time of stroke, a) to explore differences in characteristics of patients who did and did not return to work at 30 months; b) to describe the proportion of patients with paid employment and on partial or full-time sick leave over time as well as their use of employment adaptations and support; and c) to describe the course of participation in patients who did and did not remain in paid employment.

## 2 Materials and methods

### 2.1 Study design

This study was part of the Stroke Cohort Outcomes of REhabilitation (SCORE) study [14], a longitudinal cohort study that was executed from March 2014 until December 2019 at Basalt, a multidisciplinary rehabilitation facility in the Netherlands.

The Medical Ethical Committee of the University Medical Center approved the SCORE study, that is registered at the International Clinical Trial Registry Platform (https://trialsearch.who.int/Default.aspx). The current study on the long-term course of participation is reported in accordance with the STrengthening the Reporting of OBservational studies in Epidemiology (STROBE) guidelines [15].

### 2.2 Setting

In the Netherlands, after an average of 7.8 days of hospital admission for stroke, approximately 14% of the patients are referred to inpatient multidisciplinary rehabilitation in a rehabilitation facility, 15% are discharged to inpatient geriatric rehabilitation and 71% of the patients are discharged home [16]. Some of the patients who are discharged to their homes are referred to outpatient multidisciplinary rehabilitation in a rehabilitation facility [16]. As compared with geriatric rehabilitation, the population of stroke patients admitted to multidisciplinary rehabilitation is composed of the younger patients, who were more active before stroke and have complex impairments [8].

With respect to the Dutch legislation and social security system related to sick leave and work disability, it is compulsory for anyone that has paid employment with an employment contract to be insured under the Dutch Employee Insurance Schemes. This insurance obligates employers to continue to pay (a percentage of) the salary when an employee is fully or partly sick-listed during the first two years. In addition, during this period employers should do all they can to ensure that the sick employee returns to work as quickly as possible in a responsible way, including providing (temporary) modified work within the own company or elsewhere when necessary [17]. When the employee stays disabled and sick-listed for work for more than two years, the employee’s ‘ability to work’ is examined. When this ‘ability to work’ is not present anymore, the employee receives a benefit of the Dutch government and the employer is allowed to terminate the employment contract of the employee. In case of self-employment this legislation does not apply; return to work is the patient’s own responsibility, and it depends on his or her private insurance for sick leave and work disability whether or not he or she receives a benefit during sick leave if there is no ability to work.

### 2.3 Participants

All stroke patients starting with inpatient or outpatient rehabilitation at Basalt between March 2014 and December 2019 were invited by their rehabilitation physician to participate in the SCORE study when they: 1) were 18 years or older; 2) had a first or recurrent stroke less than six months ago; 3) had no dementia or psychiatric disorder; and 4) were able to complete questionnaires in Dutch. Eligible patients who were willing to participate were only included after they provided written informed consent.

The current analysis concerned a subset of patients who had paid employment at the time of stroke, were aged < 66 years (retirement age in The Netherlands in 2019) 30 months after start of rehabilitation (T30) and completed the questionnaire related to paid employment at T30.

### 2.4 Assessments

#### 2.4.1 Sociodemographic and clinical characteristics

Sociodemographic and clinical characteristics were recorded at the start of rehabilitation, i.e. baseline. Age, sex and stroke type (i.e. ischemic or haemorrhagic stroke) were extracted from the patients’ medical file. A questionnaire was used to assess educational level and living situation. Comorbidities were determined by the Dutch Life Situation Cohort Questionnaire, comprising 16 chronic diseases [18]. The Barthel Index was completed only for patients receiving inpatient rehabilitation. The Barthel Index is a nurse-reported measurement instrument that measures functional independence. It yields a score between 0 and 20, with higher scores indicating more independence [19].

#### 2.4.2 Employment prior to stroke and at follow-up

A questionnaire about paid employment prior to stroke was completed at baseline and included the following questions: type of contract (permanent, temporary, self-employed, other), amount of working hours per week according to contract, type of occupation (office job, service job or industrial/manual job) and managerial position (yes/no).

At 6 (T6), 12 (T12), 18 (T18), 24 (T24) and 30 (T30) months after baseline, patients were asked whether they had paid employment (yes/no), defined as having an employment contract or being self-employed, regardless of actually working or not (because of partial or full sick leave).

If patients indicated that they had paid employment, an additional questionnaire was completed. They were asked whether they were actually working and/or were on partial or full sick leave. This questionnaire also comprised questions on the occurrence of employment adaptations (changes of tasks, working hours, function/position, work accommodations, or a change of employer) and support related to return to work (work-related support from employer/supervisor, occupational physician, rehabilitation center or other), all over the past 6 months, in yes/no format.

#### 2.4.3 Other Patient Reported Outcome Measures (PROMs)

Besides the questionnaire concerning paid employment, the EuroQol-5 Dimensions-3 Levels (EQ-5D-3 L) [20] and four domains of the Stroke Impact Scale (SIS) version 3.0 [21] were completed at baseline. The Utrecht Scale for Evaluation of Rehabilitation-Participation (USER-P) [22] was completed at T6, T12 and T24.

The EQ-5D-3 L was used to measure health-related quality of life [20]. It comprises the following five dimensions: mobility, self-care, usual activities, pain/discomfort and anxiety/depression. Each dimension has 3 levels of severity: no problems, some problems, and extreme problems. The patient is asked to indicate his/her health state by ticking the box next to the most appropriate statement in each of the five dimensions. The resulting index ranges from –0.33 (serious problems on all five dimensions) to 1 (perfect health) [23]. Next to this index, the EQ-5D-3 L comprised a vertical visual analogue scale (VAS), ranging from 0 (‘worst imaginable health state’) to 100 (‘best imaginable health state’) to quantify the patient’s self-rated health status [20].

The SIS is a stroke-specific health status measure, that assesses several domains [21]. Items are scored on a 5-point Likert scale and transformed to a score ranging from 0–100 for each domain, with higher scores indicating better functioning on that specific domain. The domains ‘Communication’ and ‘Memory and thinking’ were administered in all patients. In April 2015, the domains ‘Mobility’ and ‘Mood and emotions’ were added.

The USER-P is a measure that is based on the International Classification of Functioning, Disability and Health (ICF) and assesses objective and subjective participation [22]. It consists of 32 items divided into three scales: Frequency, Restrictions, and Satisfaction. The Frequency scale consists of four items on vocational activities (‘paid work’, ‘unpaid work’, ‘education’, ‘household duties’), scored in hours per week ranging from 0 (not at all) to 5 (36 hours or more); and seven items on leisure and social activities, scored in frequency in the last four week ranging from 0 (not at all) to 5 (19 times or more). The Restrictions scale consists of 11 items on activities that may be restricted due to a health condition, including one item about ‘paid work, unpaid work or education’. The perceived difficulty in performing the activity is rated on a four-point scale, ranging from 0 (not possible) to 3 (without difficulty). A ‘not applicable’ option is available for every item and can be used if the item is not relevant to the patient or if experienced restrictions are not related to the patient’s health condition. The Satisfaction scale includes ten items on satisfaction with vocational, leisure and social activities and relationships. Items are rated on a scale from 0 (very dissatisfied) to 5 (very satisfied). For the items ‘paid work, unpaid work or education’ and ‘your relationship with your partner’ a ‘not applicable’ option is available. The sum score of each scale is based on all applicable items and is converted to a 0–100 scale, with higher scores indicating better participation (more time spent/higher frequency, less restrictions, higher satisfaction) [22].

### 2.5 Statistical analysis

Data analyses were performed in SPSS version 25, 2013 (IBM Corp., Armonk, NY, USA). For all statistical analyses a two-sided *p*-value of≤0.05 was considered statistically significant. Data are presented as numbers (n) with percentages (%), as means with standard deviations (SD) or as medians and interquartile ranges (IQR) depending on their nature and their distribution. The Kolmogorov-Smirnov test was used to assess whether or not continuous variables were normally distributed.

Baseline sociodemographic, clinical and employment characteristics and PROMs of included patients were compared with those of patients who had paid employment at the time of stroke and were still < 66 years old at T30, but who did not complete the questionnaire related to paid employment at T30 and were therefore excluded. For this comparison Fisher’s Exact Tests and Mann-Whitney U Tests were used. Baseline sociodemographic, clinical and employment characteristics and PROMs of patients with paid employment at T30 were compared with those of patients without paid employment at T30 using Fisher’s Exact Tests and Mann-Whitney U Tests, where appropriate.

The proportion of patients with paid employment was computed as the number of patients reporting they had paid employment at that time point divided by the number of patients completing the questionnaire on work at that time point. Only for patients with paid employment, the proportions of patients who were on sick leave, who had specific work adaptations and who received work-related support for each follow-up time point were calculated.

In order to visualize the influence of the self-reported impact of stroke on return to paid employment, patients were divided in three equal groups based on the height of their score at baseline on the SIS for each domain (i.e., Communication, Mobility, Memory and thinking, and Mood and emotions). For each tertile the percentage of patients who returned to paid employment at T30 was calculated and displayed in a bar chart.

With respect to participation, the scores of each USER-P scale at T6, T12 and T24 were compared between patients with and without paid employment using Mann Whitney U Tests. In order to make a fair comparison between patients with and without paid employment, additional analyses were done with the scores of each USER-P scale without the items concerning employment. For the Frequency scale it concerned omitting the items ‘paid work’ and ‘education’, as the latter is described as ‘only training courses taken in the context of your paid work or to help you obtain paid work’. For the Restrictions and Satisfaction scales only the item ‘paid work, unpaid work or education’ was omitted. The minimum number of completed items for the Frequency scale for the first four items was set on two instead of three, and for the Satisfaction scale this was set on five instead of six.

In addition, to evaluate whether or not USER-P scores of each scale changed over time, linear mixed models were used. Analyses were done with both the complete USER-P scale scores and the scale scores without the items related to work as dependent variables. Paid employment was included in the model as being employed at T24 (yes/no). Time was the independent variable, and also an interaction term between time and paid employment at T24 was added to the model to analyse whether the slope of the change over time was different in patients with and without paid employment.

## 3 Results

Between March 2014 and December 2019, 836 patients were included in the SCORE study. Of them, 620 (74.2%) reported whether they had paid employment or not at the time of stroke. Among these, 348 (41.6%) patients reported that they had paid employment at the time of stroke, but 288 met the inclusion criterion of being younger than 66 years old at T30. The questionnaire related to paid employment at T30 was completed by 170 (59%) of them.

These 170 patients were included in the current analyses (Table 1). Their median age was 54.2 (IQR 11.2) years and 68 (40.0%) of them were female. The included patients did not statistically significantly differ from the 118 patients who had paid employment at the time of stroke and were still younger than 66 at T30, but who did not complete the employment questionnaire at T30 (Appendix 1).

**Table 1:** Baseline characteristics of stroke patients receiving multidisciplinary rehabilitation who had paid employment at the time of stroke

		N 170	Included in the current analyses	N 86	With paid employment at T30	N 84	Without paid employment at T30	*p*-value*
Sociodemographic characteristics								
Age *in years*		170	54.2 (11.2)	86	52.7 (9.3)	84	56.4 (13.2)	0.087
Female sex		170	68 (40.0%)	86	30 (34.9%)	84	38 (45.2%)	0.210
Low education level		167	46 (27.1%)	85	20 (23.5%)	82	26 (31.7%)	0.299
Living alone		169	30 (17.8%)	86	13 (15.1%)	83	17 (20.5%)	0.423
Clinical characteristics								
Ischemic stroke		167	126 (75.4%)	84	63 (75.0%)	83	63 (75.9%)	1.000
Number of comorbidities		131	1.0 (1.0)	64	1.0 (2.0)	67	1.0 (1.0)	0.054
Barthel Index at start rehabilitation^1^		92	17.0 (9.0)	44	17.0 (8.0)	48	17.0 (9.0)	0.803
Employment characteristics prior to stroke								
Type of contract	Permanent	170	131 (77.1%)	86	64 (74.4%)	84	67 (79.8%)	**0.008**
	Temporary		12 (7.1%)		5 (5.8%)		7 (8.3%)
	Self-employed		20 (11.8%)		16 (18.6%)		4 (4.8%)
	Other		7 (4.1%)		1 (1.2%)		6 (7.1%)
Number of working hours according to contract		169	36.0 (11.0)	86	38.0 (4.0)	83	35.0 (16.0)	**0.001**
Type of occupation	Office job	155	67 (43.2%)	79	43 (54.4%)	76	24 (31.6%)	**0.014**
	Service job		51 (32.9%)		22 (27.8%)		29 (38.3%)
	Industrial or manual job		37 (23.9%)		14 (17.7%)		23 (30.3%)
	Managerial position	154	18 (11.7%)	79	13 (16.5%)	75	5 (6.7%)	0.079
Patient Reported Outcome Measures								
EQ-5D-3L index		151	0.78 (0.26)	77	0.81 (0.21)	74	0.77 (0.34)	**0.008**
EQ-5D-3L VAS		159	65.0 (26.0)	81	70.0 (20.0)	78	60.0 (31.0)	**0.003**
SIS Communication		161	92.2 (25.0)	84	96.1 (17.9)	77	89.3 (28.6)	**0.035**
SIS Mobility		84	84.7 (38.2)	39	91.7 (36.1)	45	83.3 (52.8)	0.207
SIS Memory and thinking		163	85.7 (25.0)	84	85.7 (24.1)	79	82.1 (32.1)	0.132
SIS Mood and emotions		84	79.2 (23.6)	39	80.6 (19.4)	45	75.0 (23.6)	0.183

Dichotomous variables are described as numbers with percentages (%) and continuous variables as medians with interquartile ranges; *p-values are given of Fisher Exact Tests or Mann-Whitney U Tests, when appropriate. ^1^For inpatients only. Abbreviations: EQ-5D-3L EuroQoL-5 Dimensions-3 Levels; SIS Stroke Impact Scale; VAS visual analogue scale.

### 3.1 Characteristics of patients with and without paid employment at T30

At T30, 86 patients (50.6%) reported to be in paid employment. Table 1 shows that compared to those who did not remain in the work force, patients with paid employment at T30 had statistically significantly more working hours and better EQ-5D-3 L and SIS Communication scores at baseline. In addition, they were more often self-employed (versus permanent contract *p* = 0.015; versus temporary contract *p* = 0.053; versus other *p* = 0.004) and had more often an office job (versus service job *p* = 0.026; versus industrial/manual job *p* = 0.013).

**Fig. 1:**
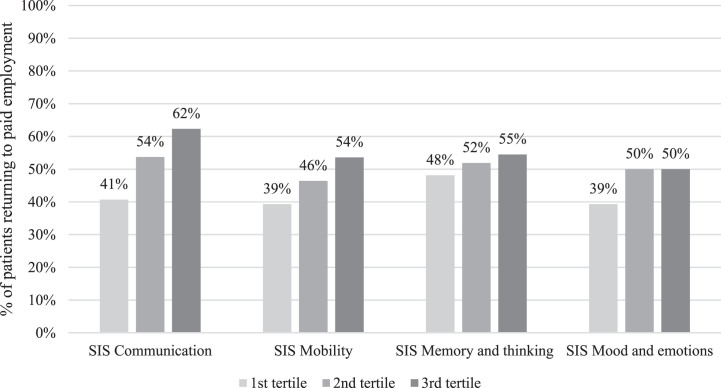
The percentage of patients returning to paid employment at 30 months after the start of the rehabilitation per tertile of the domains of the Stroke Impact Scale (SIS) at baseline. A higher score on the SIS indicates better functioning.

Figure 1 visualizes the percentage of patients who returned to paid employment at T30 for three groups of patients equal in terms of numbers representing patients with the lowest, middle and highest score for each SIS domain at baseline, respectively.

### 3.2 Paid employment over time

Table 2 shows the employment status of the 170 participants over time. The proportions of patients reporting that they were employed decreased, in particular between T18 and T24, with eventually 50.6% of patients reporting paid employment at T30. Only few patients reported changing jobs. The individual courses of patients of employment status are described in Appendix 2.

**Table 2:** Employment outcomes at all follow-up time point of patients who had paid employment at the time of stroke

	N	6 months	N	12 months	N	18 months	N	24 months	N	30 months
Employment	159		158		153		154		170
No paid employment		25 (15.7%)		25 (15.8%)		38 (24.8%)		64 (41.6%)		84 (49.4%)
Paid employment		134 (84.3%)		133 (84.2%)		115 (75.2%)		90 (58.4%)		86 (50.6%)
Presence of sick leave when employed	93		88		81		62		50
Working without sick leave		9 (9.7%)		30 (37.5%)		43 (53.1%)		50 (80.6%)		41 (82.0%)
Partial sick leave		27 (29.0%)		35 (39.8%)		21 (25.9%)		7 (11.3%)		5 (10.0%)
Full sick leave		57 (61.3%)		23 (26.1%)		17 (21.0%)		5 (8.1%)		4 (8.0%)
Employment adaptations when employed^1^	92		133		114		90		86
No employment adaptations		53 (57.6%)		67 (50.4%)		65 (57.0%)		57 (63.3%)		74 (86.0%)
Work tasks/activities		24 (26.1%)		32 (24.1%)		22 (19.3%)		13 (14.4%)		7 (8.1%)
Working hours		24 (26.1%)		38 (28.6%)		23 (20.2%)		9 (10.0%)		8 (9.3%)
Work function/position		3 (3.3%)		11 (8.3%)		9 (7.9%)		5 (5.6%)		5 (5.8%)
Work accommodations (e.g. devices)		5 (5.4%)		12 (9.0%)		6 (5.3%)		5 (5.6%)		1 (1.2%)
Change in employer		0 (0.0%)		3 (2.3%)		4 (3.5%)		3 (3.3%)		1 (1.2%)
Employment-related support^1^	92		133		114		90		86
No employment-related support		34 (37.0%)		55 (41.4%)		65 (57.0%)		64 (71.1%)		80 (93.0%)
Employer/supervisor		34 (37.0%)		53 (39.8%)		29 (25.4%)		10 (11.1%)		4 (4.7%)
Occupational physician		38 (41.3%)		55 (41.4%)		36 (31.6%)		16 (17.8%)		4 (4.7%)
Rehabilitation centre		26 (28.3%)		23 (17.3%)		4 (3.5%)		3 (3.3%)		1 (1.2%)
Other		5 (5.4%)		10 (7.5%)		15 (13.2%)		4 (4.4%)		1 (1.2%)

Variables are described as numbers with percentages (%). ^1^In the previous six months in the patients who had paid employment at that specific follow-up time point; several answers were possible when patients reported that there were employment adaptations or that they received employment-related support.

Patients reporting paid employment could also be on sick leave partially or fully. Although the proportions of patients in paid employment decreased over time, among those with paid employment, the percentage of patients reporting that they were working without sick leave increased from 9.7% at T6 to 82.0% at T30. It must be noted that at the various follow-up time points, only 58.1–70.4% of patients reporting paid employment provided information on sick leave.

### 3.3 Employment adaptations and support

Table 2 also provides insight into the implementation of employment adaptations and the support from the employer or health professionals with respect to return to work. It appeared that overall changes in tasks and activities and changes in working hours were the most frequently reported employment adaptations. With regard to support the guidance from the employer/supervisor and occupational physician were reported more often than that from the rehabilitation center or other sources. Like the questions on sick leave, the response rates to the questions on employment adaptations and support at the various time points were varying between 68.7–100.0%.

### 3.4 Participation over time

Table 3 shows the scores of all three USER-P scales of the total group of patients and separately for patients either reporting or not reporting paid employment at T6, T12 and T24.

Regarding the differences of USER-P scale scores between patients with and without paid employment, there were no statistically significant differences at T6, whereas at T12 patients reporting paid employment had significantly better scores for the USER-P Frequency and Restrictions scales (*p* < 0.05) and at T24 for all three USER-P scales (all *p* < 0.001). With respect to USER-P scale scores over time, there were no statistically significant changes over time, neither in the total, nor within the subgroups of patients with or without paid employment at T24 (Appendix 3).

**Table 3:** USER-P in stroke patients with and without paid employment up to 24 months after the start of rehabilitation

	N	6 months	*p*-value*	N	12 months	*p*-value*	N	24 months	*p*-value*
USER-P Frequency
*All items*
All patients	159	33.6 (16.4)		160	33.6 (14.8)		158	31.4 (16.3)
Paid employment	131	34.6 (17.5)	0.182	133	34.6 (15.0)	**0.001**	90	36.0 (17.0)	**<0.001**
No paid employment	25	29.2 (13.0)		25	28.9 (16.3)		61	29.3 (10.4)
*Without item* ‘*Paid work*’ *and education*’									
All patients	159	34.3 (15.7)		160	33.1 (14.8)		158	32.9 (16.8)
Paid employment	131	34.3 (16.4)	0.470	133	33.6 (14.3)	0.799	90	31.8 (17.9)	0.697
No paid employment	25	33.6 (17.3)		25	33.3 (23.6)		61	34.3 (13.6)
USER-P Restrictions
*All items*
All patients	161	83.3 (33.0)		157	87.5 (27.3)		158	87.9 (33.3)
Paid employment	133	83.3 (33.2)	0.197	130	89.4 (27.9)	**0.005**	90	96.8 (19.0)	**<0.001**
No paid employment	25	76.7 (43.3)		25	74.1 (35.3)		61	70.0 (30.8)
*Without item* ‘*Paid work, unpaid work or education*’									
All patients	161	86.7 (33.3)		157	90.0 (30.0)		158	90.0 (29.3)
Paid employment	133	88.9 (33.3)	0.284	130	92.6 (26.7)	**0.032**	90	96.7 (18.5)	**<0.001**
No paid employment	25	80.0 (42.1)		25	74.1 (34.6)		61	73.3 (29.6)
USER-P Satisfaction
*All items*
All patients	158	72.2 (27.1)		157	72.5 (25.8)		155	72.5 (27.5)
Paid employment	130	72.2 (26.9)	0.713	130	72.5 (26.3)	0.093	90	77.6 (26.5)	**<0.001**
No paid employment	25	75.0 (33.8)		25	69.4 (29.5)		58	65.6 (27.8)
*Without item* ‘*Paid work, unpaid work or education*’									
All patients	158	75.0 (25.7)		158	75.0 (25.3)		158	75.0 (26.2)
Paid employment	130	75.0 (25.0)	0.813	131	75.0 (26.7)	0.064	90	77.8 (26.9)	**<0.001**
No paid employment	25	75.0 (32.5)		25	69.4 (29.3)		61	69.4 (26.4)

Variables are described as medians with interquartile ranges; *p-values are given of Mann-Whitney U Tests comparing patients with and without paid employment. Abbreviations: USER-P Utrecht Scale for Evaluation of Rehabilitation–Participation.

When leaving out the items concerning employment, again at T6 no statistically significant differences in USER-P scale scores were seen between patients who did and did not report paid employment at that time point. At T12 only the difference for the Restrictions scale remained. At T24, the scores for the Restrictions and Satisfaction scales were statistically significantly better for patients with paid employment, whereas the Frequency scale score was not statistically significantly different. Regarding the course of the Frequency scale score, its scores diminished over time for patients with paid employment (β –1.74, 95% CI –2.96 ––0.52, *p* = 0.005), but not in patients without paid employment at 24 months.

## 4 Discussion

This study on the long-term course of employment outcomes and overall participation in patients with paid employment pre-stroke receiving multidisciplinary rehabilitation, found that half of them reported paid employment at 30 months after starting rehabilitation. The proportion of patients that had paid work was highest at six months with a marked decrease between 18 and 24 months after start of rehabilitation. These results reflect the Dutch social security system, where patients who are employed but sick-listed are entitled to a two-year period of (partial) salary payment and possible re-integration.

Baseline characteristics of employment, namely self-employment, a higher number of working hours and having an office job, were associated with having paid employment at T30. In addition, the patients remaining in the work force reported better quality of life and less impact of their stroke on communication at baseline.

With respect to participation that is not employment related, patients who reported paid employment experienced less restrictions and were more satisfied than patients who did not. However, frequencies of participation outside of employment did not differ and decreased with time in those who retained work.

Our study showed a decrease in proportions of patients reporting paid employment that seems in contrast to previous studies such as that of Saeki et al. [24], that demonstrate an increase of patients that return to work over time. Nevertheless, this contrast is not an actual contrast, because when looking at the proportions of patients that reported paid employment and were actually working, the same increase over time is seen.

In our study, half of the patients returned to paid employment at 30 months, but it is difficult to directly compare this result with previous studies, in part due to methodological differences. Therefore, and as mentioned in the introduction, estimated proportions of stroke patients returning to work varied largely [9–12]. Nevertheless, our finding is in the same range as the proportions seen in a previous Dutch cross-sectional, hospital-based study including patients aged 18–65 years at 2–5 years post-stroke, where 39% returned to work [25]. The patients of that study were younger and more often had an ischemic stroke than the patients in our study, but the proportions females and patients with a low level of education were comparable. In addition, our results were in the same range of a review which calculated a pooled summary estimate of return to work two years post-stroke of 67.4% [9]. Overall, the heterogeneity in study methodology seen in the studies on this topic underlines the need for international consensus on how to best define and assess employment status in clinical and epidemiological studies in stroke patients [10, 26, 27].

This quantitative study did not elaborate on why patients were not able to return to paid employment. A previous qualitative study found that return to paid employment was influenced not only by the direct impact of stroke (stigma and discrimination, degree of impairments, ability to engage in activities in the community and work related tasks), but also by the realignment of life priorities (recovery was more important than work, the value of work, new lifestyle after stroke and responsibilities towards self and others) and engagement with support and resources (barriers, support from employers, colleagues and family and explore potential job opportunities) [28]. Therefore, depending on the patient’s health status, the work situation, and the social security system, the work status of patients may vary largely within and across patients, with possible combinations of either or not working fully or partially and either or not being on fulltime or parttime sick leave, and either or not receiving a fulltime or parttime disability pension. For a detailed description an individual interview or an extensive questionnaire is needed.

Regarding the association of baseline characteristics with long-term paid employment, our findings are in general in line with previous literature. Regarding work characteristics, previous literature in particular demonstrated that white collar occupation was beneficial for return to work compared to blue collar occupation [24, 29]. Our study found that self-employment and more working hours at baseline were also associated with return to work. It could be hypothesized that these patients were the breadwinners and therefore the need to return to paid employment was high. This is supported by previous research which found that 90.9% of stroke patients returning to work were the breadwinner of their family [30].

Regarding stroke characteristics, previous studies found that the presence of aphasia was negatively associated with return to work [29, 31], which is in line with the observation in our study that the SIS Communication score was lower in patients who did not return to work.

Moreover, it has been found previously that normal muscle strength, absence of apraxia and more independence in activities of daily life measured with the Barthel Index were positively related to return to work [24, 31], while other studies showed no influence of stroke severity [32]. In our study, better scores for the EQ-5D-3 L, which involves questions about mobility and activities of daily life, were associated with paid employment at T30. However, this was not true for the SIS Mobility nor for the Barthel Index, perhaps because of low number of patients for whom these outcomes were known.

This study found that at all time points, a considerable proportion of patients reported employment adaptions in the previous six months, with only few changing jobs. The need for reductions in working hours and employment modifications because of changes in abilities due to stroke are previously mentioned in literature [10]. However, our results are hard to compare with those from other studies, as we did not record the cumulative, overall changes from baseline onwards. However, by recording adaptations over the previous six months, we were able to demonstrate that the occurrence of adaptations in those with paid employment decreased with time. It remains unclear to what extent this finding can be interpreted as a decreasing need and successful work integration over time.

Support from the employer and occupational physician were the most often reported sources of help. Although we have no cumulative figures, the findings at six months can be interpreted on their own, where it is striking that less than half of the patients reported support from their employer or occupational physician. These results may be flattened by the reporting of self-employed patients, but nevertheless may indicate that there is room for improvement, in particular given the far-reaching legal responsibility to support the return to work process in the Netherlands [26]. Work-directed interventions in combination with education/coaching were shown previously to be effective regarding return to work [33]. It could be considered to include these interventions more consistently by the employer, occupational physician or rehabilitation center.

Considering participation outside of paid employment, it was striking that although frequencies were comparable, patients without paid employment experience more restrictions and less satisfaction with this participation. A previous study demonstrated that stroke patients retain predominately more sedentary and home-based activities and fewer physically demanding and community-based activities [34]. This might be more the case for patients without paid employment, explaining the difference in restrictions and satisfaction. Indeed, a need for well-founded, proven effective interventions for achieving meaningful participation outside of employment has been mentioned previously [35]. It was suggested that this might require different types of support at various stages after stroke [36] and should take into account the social support system and other environmental factors, such as transportation [37, 38].

### 4.1 Strengths and limitations

A strength of this study is the long-term, prospective design and the comprehensive assessment of both employment and participation. The computation of the USER-P scale scores with and without work-related items allowed a fair comparison on the perception of participation of patients who remained in paid employment and who did not. Limitations include the relatively small sample size, inclusion from only one rehabilitation facility and missing items in questionnaires of patients in the study. Moreover, the study population was selected based on their completion of the 30-months assessment. Although their characteristics at baseline did not differ from those who did not complete the study, selection bias cannot be ruled out. Another limitation was that the definition of 'having paid employment' could reflect different situations, including being actually at work or being fully or partly sick-listed. Although we aimed to gather detailed information from all patients, the precise working situation was missing for a proportion of patients. Finally, the results from the present study are influenced by the Dutch context and might therefore not be applicable to other countries with different legislation, social security and health systems.

## 5 Conclusion

The results of the present study suggest that there are windows of opportunity to improve the participation outcomes for patients in paid employment at the time of stroke receiving rehabilitation, both in those who do and do not remain in the work force, by implementing more consistently effective work-directed interventions and interventions for achieving meaningful participation outside of employment.

## Ethical approval

The Medical Ethical Committee of the University Medical Center approved the SCORE study (NL465321.058.13).

## Informed consent

Eligible patients who were willing to participate were only included after they provided written informed consent.

## Conflict of interest

The authors declare that they have no conflicts of interest.

## Supplementary Material

Supplementary Material
