# Supplementary material for: Stroke survivors’ long-term participation in paid employment
Source: Work. 2024 Mar 8;77(3):839–50. doi: 10.3233/WOR-230037 (PMC10977381; doi:10.3233/WOR-230037)
Supplement: Supplementary Material [file wor-77-wor230037-s001.docx]

**Appendix 1. Baseline characteristics of stroke patients receiving multidisciplinary rehabilitation who had paid employment at the time of stroke**

|  | | | **N**  **170** | **Included in the current analyses** | **N**  **118** | **Excluded in the current analyses** | ***p*-value*** |
| --- | --- | --- | --- | --- | --- | --- | --- |
| **Sociodemographic characteristics** | | |  |  |  |  |  |
| Age *in years* | | | 169 | 54.2 (11.2) | 115 | 53.9 (13.2) | 0.839 |
| Female sex | | | 170 | 68 (40.0%) | 118 | 51 (43.2%) | 0.627 |
| Low education level | | | 167 | 46 (27.5%) | 116 | 43 (37.1%) | 0.093 |
| Living alone | | | 169 | 30 (17.8%) | 115 | 29 (25.2%) | 0.138 |
| **Clinical characteristics** | | |  |  |  |  |  |
| Ischemic stroke | | | 167 | 126 (75.4%) | 117 | 90 (76.9%) | 0.888 |
| Number of comorbidities | | | 131 | 1.0 (1.0) | 97 | 1.0 (2.0) | 0.832 |
| Barthel Index at start rehabilitation^1^ | | | 92 | 17.0 (9.0) | 70 | 17.0 (9.0) | 0.494 |
| **Employment characteristics** | | |  |  |  |  |  |
| Type of contract | | Permanent | 170 | 131 (77.1%) | 118 | 84 (71.2%) | 0.668 |
|  |  | Temporary |  | 12 (7.1%) |  | 11 (9.3%) |  |
|  |  | Self-employed |  | 20 (11.8%) |  | 16 (13.6%) |  |
|  |  | Other |  | 7 (4.1%) |  | 7 (5.9%) |  |
| Number of working hours according to contract | | | 169 | 36.0 (11.0) | 116 | 36.0 (16.0) | 0.119 |
| Type of occupation | Office job | | 155 | 67 (43.2%) | 97 | 37 (38.1%) | 0.219 |
|  | Service job | |  | 51 (32.9%) |  | 27 (27.8%) |  |
|  | Industrial or manual job | |  | 37 (23.9%) |  | 33 (34.0%) |  |
| Managerial position | | | 154 | 18 (11.7%) | 98 | 11 (11.2%) | 1.000 |
| **Patient Reported Outcome Measures** | | |  |  |  |  |  |
| EQ-5D-3L index | | | 151 | 0.78 (0.26) | 108 | 0.76 (0.35) | 0.191 |
| EQ-5D-3L VAS | | | 159 | 65.0 (26.0) | 108 | 64.0 (25.0) | 0.528 |
| SIS Communication | | | 161 | 92.2 (25.0) | 109 | 89.3 (26.8) | 0.245 |
| SIS Mobility^2^ | | | 84 | 84.7 (38.2) | 80 | 86.1 (29.9) | 0.492 |
| SIS Memory and thinking | | | 163 | 85.7 (25.0) | 111 | 78.6 (35.7) | 0.270 |
| SIS Mood and emotions | | | 84 | 79.2 (23.6) | 82 | 77.8 (22.2) | 0.526 |

Dichotomous variables are described as numbers with percentages (%) and continuous variables as medians with interquartile ranges; **p*-values are given of Fisher Exact Tests or Mann-Whitney U Tests, when appropriate.

^1^For inpatients only

^2^Added later to the set of questionnaires

Abbreviations: EQ-5D-3L EuroQoL-5 Dimensions-3 Levels; SIS Stroke Impact Scale; VAS visual analogue scale.

**Appendix 2. Overview of employment status at the different measurement moments of stroke patients with paired measurements.**

|  | **N**  **126** | **6 months** | **12 months** | **18 months** | **24 months** | **30 months** |
| --- | --- | --- | --- | --- | --- | --- |
| **Group 1 Paid employment at all measurement moments** | | | | | |  |
|  | 54 (42.9%) | + | + | + | + | + |
| **Group 2 Paid employment over time** | | | | | |  |
| Paid employment at 12, 18, 24 and 30 months | 5 (4.0%) | - | + | + | + | + |
| Paid employment at 18, 24 and 30 months | 0 (0.0%) | - | - | + | + | + |
| Paid employment at 24 and 30 months | 2 (1.6%) | - | - | - | + | + |
| Paid employment at 30 months | 0 (0.0%) | - | - | - | - | + |
| **Group 3 No longer paid employment over time** | | | | | |  |
| No longer paid employment at 12, 18, 24 and 30 months | 6 (4.8%) | + | - | - | - | - |
| No longer paid employment at 18, 24 and 30 months | 9 (7.1%) | + | + | - | - | - |
| No longer paid employment at 24 and 30 months | 20 (15.9%) | + | + | + | - | - |
| No longer paid employment at 30 months | 12 (9.5%) | + | + | + | + | - |
| **Group 4 Having paid employment fluctuates over time** | | | | | |  |
| Paid employment at 6, 18, 24 and 30 months | 1 (0.8%) | + | - | + | + | + |
| Paid employment at 6, 12, 24 and 30 months | 4 (3.2%) | + | + | - | + | + |
| Paid employment at 6, 12, 18 and 30 months | 1 (0.8%) | + | + | + | - | + |
| Paid employment at 6 and 18 months | 1 (0.8%) | + | - | + | - | - |
| Paid employment at 12 months | 2 (1.6%) | - | + | - | - | - |
| **Group 5 No longer paid employment after baseline** | | | | | |  |
|  | 9 (7.1%) | - | - | - | - | - |

+ means paid employment at that measurement moment

- means no paid employment at that measurement moment

**Appendix 3. Linear mixed model results of USER-P scales over time**

|  | **Complete USER-P scale** | | | **USER-P scale without items paid employment** | | |
| --- | --- | --- | --- | --- | --- | --- |
|  | **β** | **95% CI** | ***p-*value** | **β** | **95% CI** | ***p-*value** |
| **USER-P Frequency scale** | | | | | | |
| Time | -0.62 | -1.97 – 0.74 | 0.370 | 0.45 | -1.03 – 1.93 | 0.550 |
| Paid employment at T24 | 7.81 | 3.31 – 12.31 | 0.001 | 6.77 | 1.82 – 11.71 | 0.007 |
| Time*Paid employment at T24 | 0.52 | -1.23 – 2.28 | 0.557 | -2.19 | - 4.11 – -0.27 | 0.026 |
| **USER-P Restrictions scale** | | | | | | |
| Time | 1.19 | -0.87 – 3.26 | 0.257 | 1.41 | -0.59 – 3.41 | 0.166 |
| Paid employment at T24 | 16.54 | 9.28 – 23.80 | <0.001 | 15.24 | 8.16 – 22.32 | *<*0.001 |
| Time*Paid employment at T24 | 0.60 | -2.08 – 3.29 | 0.659 | -0.19 | -2.78 – 2.41 | 0.888 |
| **USER-P Satisfaction scale** | | | | | | |
| Time | 0.45 | -1.50 – 2.39 | 0.652 | 0.09 | -1.84 – 2.02 | 0.930 |
| Paid employment at T24 | 12.78 | 5.48 – 20.07 | 0.001 | 12.44 | 5.12 – 19.77 | 0.001 |
| Time*Paid employment at T24 | 0.31 | -2.17 – 2.80 | 0.804 | 0.10 | -2.38 – 2.59 | 0.935 |

Abbreviations: CI confidence interval; T24 time measurement point 24 months after start of the rehabilitation; USER-P Utrecht Scale for Evaluation of Rehabilitation–Participation.
